# The efficacy and safety of the herbal medicine *geonchildan* for patients with active rheumatoid arthritis: study protocol for a randomized, double-blind, placebo-controlled, parallel pilot trial

**DOI:** 10.1186/s13063-018-2849-3

**Published:** 2018-09-03

**Authors:** Seunghoon Lee, Yeeun Cho, Jihye Kim, Jung Won Kang, Ga Young Yoon, Jun-Hwan Lee, So-Young Jung, Ojin Kwon, Kyung-Min Shin, Jae-Dong Lee

**Affiliations:** 1 Department of Acupuncture & Moxibustion Medicine, Kyung Hee University Korean Medicine Hospital, 23 Kyunghee dae-ro, Dongdaemun-gu, Seoul, 02447 South Korea; 2 Department of Clinical Korean Medicine, Graduate School, Kyung Hee University, Seoul, South Korea; 3 Department of Acupuncture & Moxibustion, College of Korean Medicine, Kyung Hee University, Seoul, South Korea; 4 Clinical Medicine Division, Korea Institute of Oriental Medicine, Daejeon, 34054 South Korea; 5 University of Science & Technology (UST), Korean Medicine Life Science, Campus of Korea Institute of Oriental Medicine, Daejeon, 34054 South Korea

**Keywords:** Geonchildan, Rheumatoid arthritis, Herbal medicine, Randomized clinical trial, Study protocol

## Abstract

**Background:**

This study aims to assess the efficacy and safety of *geonchildan*, a Korean traditional herbal medicine, for patients with active rheumatoid arthritis (RA) and evaluate the feasibility of a large-scale confirmatory clinical trial.

**Methods/design:**

This is a randomized, double-blind, placebo-controlled, parallel two-arm pilot trial in Seoul, Korea. Altogether, 30 patients diagnosed with RA for at least 3 months and with a Disease Activity Score for 28 joints (DAS28) ≥ 3.2 will be enrolled. Participants are randomly assigned to one of two groups, the experimental group or the placebo group, in a 1:1 ratio and will make four scheduled visits. The participants will be administered *geonchildan* or a placebo three times per day for 12 weeks. The change in DAS28 will be examined as the primary efficacy outcome. The secondary efficacy outcomes include the proportion of patients achieving ACR20, ACR50, ACR70, and EULAR responses; the DAS28 sub-items; the consumption of medication; Korean Health Assessment Questionnaire scores; inflammatory parameters; and the Korean medical diagnostic pattern indicator. Adverse events and laboratory test results will be recorded to evaluate safety. The process, resources used, and management of the study will also be assessed to determine the feasibility of a large-scale trial.

**Discussion:**

This is the first clinical trial to explore the efficacy and safety of *geonchildan* in patients with active RA. If the superiority of *geonchildan* versus the placebo is demonstrated and the study design is feasible, this study could form the foundation for a large-scale clinical trial. The results will be published in a peer-reviewed journal.

**Trial registration:**

Clinical Research Information Service, KCT0001943. Registered on 14 June 2016.

**Electronic supplementary material:**

The online version of this article (10.1186/s13063-018-2849-3) contains supplementary material, which is available to authorized users.

## Background

Rheumatoid arthritis (RA) is the most common autoimmune inflammatory disease. It affects 0.5 to 1.0% of the population worldwide, causing chronically progressive inflammation [1]. It is characterized by severe joint damage, loss of physical function, distress, and poor quality of life. Whilst the early use of disease-modifying antirheumatic drugs (DMARDs), such as methotrexate, is recommended to achieve complete or long-lasting remission, a substantial number of patients experience lasting disease activity and require additional DMARDs or biological drugs [2]. These drugs are known to induce a considerable number of adverse events (AEs), even life threatening ones, which has led an increasing number of patients with RA to seek complementary and integrative medicine, including herbal medicines [3].

Several randomized controlled trials (RCTs) have been conducted to identify the efficacy and safety of herbal medicines for treating RA, but confirmatory conclusions have not been drawn due to methodological flaws and inadequate reporting [1]. Extracts of *Tripterygium wilfordii*, commonly known as thunder god vine, are among the most well-known herbal medicines used for RA in China and researched in China. Several studies have shown that these may reduce some RA symptoms compared to a placebo [4–7]. However, the usefulness is controversial because the oral use of these extracts may be associated with several side effects [1].

*Geonchildan* is a herbal decoction of *Rhus verniciflua* Stokes. This plant in the *Anacardiaceae* family has long been used as an ingredient in arthritis medication in East Asian traditional medicine [8]. Some experimental studies have reported that *Rhus verniciflua* Stokes has marked antioxidant and anti-inflammatory effects, supporting its use for RA [9–11]. In particular, a recent study showed that the flavonol-rich *Rhus verniciflua* Stokes and its major compound, fisetin, suppress some inflammatory cytokines and chemokines and also angiogenic factors in IL-1β-stimulated RA fibroblast-like synovial cells and inflammation-related mouse models [11].

In Kyung Hee University Korean Medicine Hospital, *geonchildan* has been prescribed to patients with RA since 2011. However, no prospective clinical trials have yet evaluated the effects of *geonchildan* for patients with RA. Therefore, we have designed a randomized, placebo-controlled, double-blind, exploratory pilot clinical trial to assess the efficacy and safety of *geonchildan* in the treatment of patients with RA and to evaluate the feasibility of a large-scale confirmatory clinical trial.

## Methods/design

### Objectives

The primary aim of this study is to determine if *geonchildan* is superior to placebo at decreasing the Disease Activity Score for 28 joints (DAS28) from baseline after 12 weeks of treatment. The secondary outcomes of this study are as follows:

1. proportion of patients achieving ACR20, ACR50, ACR70, and European League Against Rheumatism (EULAR) responses
2. DAS28 sub-items
3. consumption of medication
4. Korean Health Assessment Questionnaire (KHAQ) scores
5. inflammatory parameters
6. Korean medical diagnostic pattern indicator (12-item Blood Stasis Questionnaire or BSQ)

We hypothesize that these outcomes will improve more significantly in the group receiving *geonchildan* than in the group receiving the placebo after 12 weeks of treatment and that *geonchildan* is a safe treatment as verified using blood tests, vital signs, chest radiography, and electrocardiography (ECG). This study design will be assessed to see if the process, resources used, and management are feasible for a large-scale confirmatory trial.

### Design and setting

This study is a randomized, double-blind, placebo-controlled, parallel, pilot clinical trial. After voluntarily signing informed consent, participants who are eligible for this study are assigned randomly to one of two groups (the experimental group or the placebo group) with a 1:1 allocation ratio. Patients will be administered one capsule of *geonchildan* or placebo three times per day for 12 weeks.

#### Recruitment strategy

The participants will be recruited from the Acupuncture and Moxibustion Department of Kyung Hee University Korean Medicine Hospital in Seoul, Korea. A total of 30 patients with RA will be recruited through publicity such as broadcast media, newspapers, the hospital’s homepage, and advertisements in nearby welfare centers and subway stations. Screening will continue until 30 participants have been enrolled. The estimated recruitment period is from January 2017 to July 2018.

#### Study plan

**Fig. 1:**
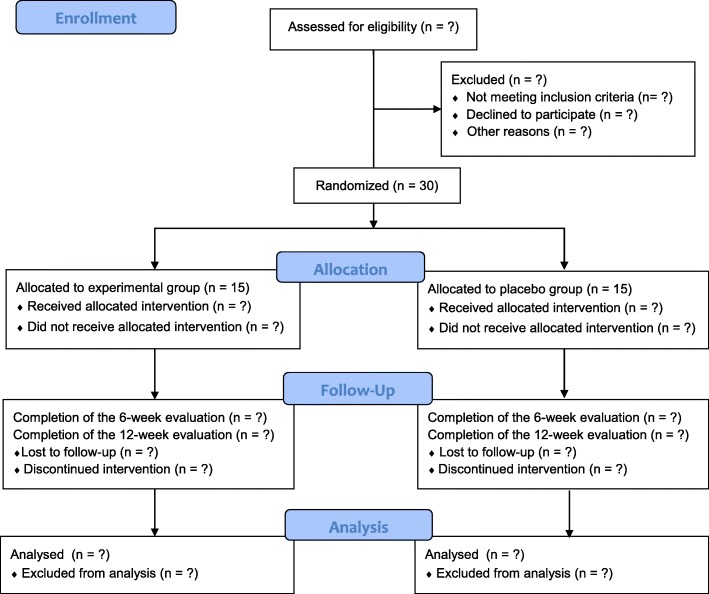
A flow chart of the study process

After participants have consented voluntarily to the study at the first visit, they will be screened using the survey measures, blood and urine tests, chest radiography, and ECG. The results of the tests will be conveyed by telephone to them and patients who meet the inclusion criteria will be requested to visit for randomization within 2 weeks of the screening visit. Patients who use nonsteroidal anti-inflammatory drugs (NSAIDs) for other diseases will be eligible to participate in the study after a 2-week washout period. On the second visit, patients will be randomly assigned to one of the two groups and will be provided with the investigational drugs. Participants will be administered one capsule of *geonchildan* or placebo three times per day for 12 weeks. During the 12 weeks of treatment, participants will be free to continue with their standard treatment with methotrexate, NSAIDs, or steroids, without modification. Moreover, one additional visit will be scheduled 6 weeks after randomization. A total of three visits will take place during the treatment period. A flow chart of the study process is shown in Fig. 1. This trial protocol was developed according to the Standardized Protocol Items: Recommendations for Interventional Trials (SPIRIT) guidelines (see Additional file 1) [12].

### Types of participants

#### Inclusion criteria

Participants who meet the following conditions will be included:

1. Men and women aged 19 to 75 years
2. Patients who have been diagnosed with RA according to the 2010 ACR/EULAR classification criteria and have had RA for at least 3 months prior to enrolment
3. Patients with active RA (DAS28 ≥ 3.2)
4. Patients who have taken methotrexate for more than 3 months; the dose of methotrexate should have been maintained for at least 8 weeks before participation in the trial (7.5–20 mg/week oral or equivalent injection dose)
5. Patients with at least three swollen joints and three tender joints

To participate, patients must provide informed consent in writing after being informed of the purpose and characteristics of the study.

#### Exclusion criteria

Participants will be excluded if any of the following conditions are satisfied:

1. Patients with at least one of the following laboratory abnormalities at screening
  White blood cell count ≤3000/mm^3^
  Hemoglobin < 8.5 g/dL
  Platelet count < 100,000/mm^3^
  Serum creatinine > 2.0 mg/dL
  Aspartate transaminase or alanine transaminase at least twice the upper limit of the normal range
2. Patients who have had or have planned a surgical procedure for RA.
3. Patients with severe infections including major respiratory diseases or who have received systematic antibiotics within 2 weeks
4. Patients with clinically uncontrolled cardiovascular disease
5. Patients with a history of malignancy within 5 years (however, patients who have a history of completely treated nonmelanoma skin cancer or uterine cervical cancer may be included)
6. Patients with autoimmune diseases other than RA
7. Patients with a history of severe hypersensitivity or allergic reaction to the investigative drug or *Rhus verniciflua* Stokes (e.g., poison ivy dermatitis)
8. Patients who have participated in other clinical studies within 3 months of screening
9. Patients with medical or psychological contraindications for the investigative drug
10. Patients who are pregnant, plan to become pregnant, or will be breast-feeding during the clinical trial
11. Patients with difficulty moving voluntarily, with high disease activity, or who are unsuitable for study inclusion in the investigator’s judgment
12. Patients who have received a steroid injection into the glenoid cavity of the evaluation joint within 4 weeks of screening
13. Patients who have received oral steroids of more 10 mg per day, have newly started treatment within 4 weeks of screening, or have changed their dose of steroids within 2 weeks of screening
14. Patients who have newly started treatment with NSAIDs within 4 weeks of screening or have changed their dose of NSAIDs within 2 weeks of screening.
15. Patients who have received biological medicine or cell proliferation inhibitors before screening
16. Patients who have received DMARDs except methotrexate before screening
17. Patients who are being treated for liver or kidney disease

### Randomization and allocation concealment

An independent, blinded statistician at the Korea Institute of Oriental Medicine will generate random numbers using the stratified block randomization method in SAS version 9.4 (SAS institute Inc., Cary, NC). Altogether, 30 eligible participants will be assigned to each group in a 1:1 ratio. The randomization will be stratified according to the 12-item BSQ (score <3 or score ≥3). The block size will be concealed to all investigators and sequentially numbered. Sealed opaque envelopes will be used to conceal group allocation and to avoid selection bias. Allocation concealment will be maintained throughout the study, since patients will be given identically packaged, consecutively numbered drug containers.

### Blinding

The participants, outcome assessors, study monitors, data managers, and statisticians will be blinded to the allocation. If possible, participants will be advised not to compare their investigational drugs with each other. The allocation guessing for blinding test will be conducted at the last visit by participants. They will choose one of the following three options based on their personal feeling about the drug they received: 'real drug', 'placebo drug', or 'do not know. The blinding will be maintained until all 30 participants have completed the study, the data analysis is complete, and the database is locked.

Separate emergency codes will be opened only if the researchers must inform a participant of which investigational drugs they are receiving (e.g., to determine the subsequent treatment to manage a serious AE).

### Intervention

The total dose of each capsule of *geonchildan* is 447 mg, of which 200 mg is a dried herbal extract. The herbal extract was obtained from the cortex of *Rhus verniciflua* Stokes. The plant material and the sample were deposited in the herbarium of the Quality Control Department of Hanpoong Pharm and Foods Co., Ltd. (Wanju-Gun, South Korea). The raw materials were extracted with water at 90–100 °C for 2 h and filtered. The process was repeated four times. The filtrate was vacuum-dried to yield *geonchildan* extract (yield, 5.0%). A high-performance liquid chromatography analysis was performed to quantify the chemical constituents of the herbal extract. The herbal extract of *geonchildan* was controlled so that it contains more than 4.99 mg/g of fisetin (C_15_H_10_O_6_) and less than 5 ppm of urushiol. To test the purity, heavy metal tests, residual pesticide tests, and a microbial limit test were conducted according to the Korea Pharmacopeia. The items tested for included total heavy metals, lead (Pb), arsenic (As), dichlorodiphenyltrichloroethane, benzene hexachloride, aldrin, dieldrin, endrin, total number of aerobic microorganisms, total number of fungi, *Escherichia coli*, *Salmonella*, *Pseudomonas aeruginosa*, and *Staphylococcus aureus*.

The placebo capsules that will be used in the trial are filled with a powder that resembles the herbal extract. The powder is made of lactose hydrate, colored with caramel, and flavored with a ginseng-like fragrance. The *geonchildan* and placebo capsules are manufactured so that there is no difference in shape, color, size, odor, or taste. All manufacturing processes for the *geonchildan* and placebo capsules were conducted at Hanpoong Pharm and Foods Co., Ltd., according to the Korean Good Manufacturing Practices.

Participants will ingest one capsule of *geonchildan* or placebo, three times per day for 12 weeks. This dosage regimen was determined after a clinical chart review (data not shown). During the 12 weeks of treatment, participants will comply with the study protocol by visiting the clinic every 6 weeks and undergoing a scheduled examination. If AEs due to the investigational drugs occur, the researchers will alter the dose or frequency according to internal guidelines.

#### Permitted concomitant treatments

Methotrexate administered for more than 3 months with the dose maintained for at least 8 weeks will be allowed without a dose change during the study period. Oral steroids (less than 10 mg per day) and NSAIDs started for at least 4 weeks with the dose maintained for at least 2 weeks will be permitted and any change of dose will be recorded. Other drugs that are unrelated to RA will be permitted if they have been administered for more than 30 days before the screening visit. Participants who use NSAIDs and do not satisfy the above-mentioned criteria can participate after a 2-week washout period.

#### Prohibited concomitant treatments

Prohibited drugs include agents that can affect RA and the primary outcome, such as (1) DMARDs except methotrexate, (2) steroid injections, (3) oral steroids at a dose higher than 10 mg per day or administered for other diseases, (4) NSAIDs administered for other diseases, and (5) biological medicines, cell proliferation inhibitors, or immunosuppressants.

### Outcomes

**Fig. 2:**
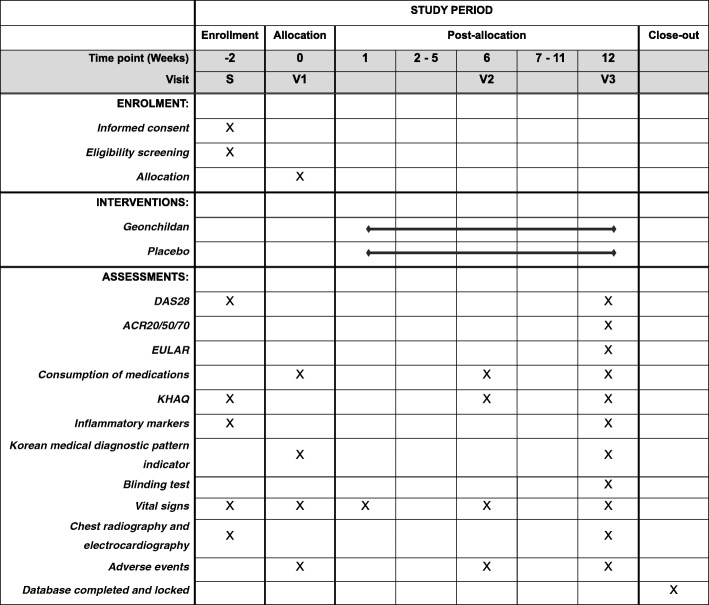
Schedule of enrolment, interventions, and assessments. ACR American College of Rheumatology, DAS28 Disease Activity Score for 28 joints, EULAR European League Against Rheumatism, KHAQ Korean Health Assessment Questionnaire, S screening visit, V1 visit 1, V2 visit 2, V3 visit 3

The details of the outcome measures and time schedule are shown in Fig. 2 [12].

#### Primary outcome measurement

Changes in DAS28 based on erythrocyte sedimentation rate (ESR) scores will be used as the primary outcome measurement. The DAS28 is a validated and widely accepted measure for RA disease activity and consists of four components: number of tender joints out of 28, number of swollen joints out of 28, C-reactive protein or ESR, and the patient’s global assessment of disease activity on a visual analogue scale (VAS) (0–100 mm). Scores of 2.6 to ≤3.2, <3.2 to ≤5.1, and >5.1 represent low, moderate, and high disease activity, respectively [13, 14]. Remission is commonly considered to be DAS28 < 2.6 [13]. A change in DAS28 score of 1.2 is considered to be the minimal clinically important difference [15]. The separate sub-items of DAS28 will also be analyzed.

#### Secondary outcomes

The proportion of patients achieving ACR20, ACR50, and ACR70 at week 12 after randomization will be used as a secondary outcome measurement. According to the criteria, ACR20 is defined as an improvement in both tender and swollen joints by 20% or more and improvement of 20% or more in three or more of the following five items: the patient’s assessment of pain on a VAS, the evaluator’s assessment of global health status on a VAS, the patient’s assessment of global health status on a VAS, the KHAQ score, and laboratory tests (ESR or C-reactive protein level). When the degree of improvement is 50% or higher and 70% or higher, it is defined as ACR50 and ACR70, respectively [16, 17].

##### European League against Rheumatism response

The proportion of EULAR response (disease remission, good, or moderate) will also be measured. Based on the DAS, the disease activity is classified according to the change in the score over time. If it is classified as good or moderate, it is regarded as an EULAR response [18].

##### Consumption of medications

Any changes in the dosage of oral corticosteroids and NSAIDs will be recorded in a case report form. The drug name, total daily dose, and single dose should be record at randomization and at 6 and 12 weeks. Oral steroids at a dose less than 10 mg per day will be allowed.

##### Korean health assessment questionnaire

HAQ, developed by Fires et al., is a tool that assesses the health status of patients with arthritis. It is a disease-specific health status instrument for RA. In this study, we will use the KHAQ, which has undergone a cross-cultural adaptation and confirmation process [19, 20]. The KHAQ consists of 20 questions divided into eight areas: (1) dressing and grooming, (2) rising, (3) eating, (4) walking, (5) hygiene, (6) reaching out, (7) holding hands, and (8) activities. A high score signifies that the disability in daily living activities is severe [20].

##### Inflammatory markers

Blood from each patient before and after treatment will be sampled to investigate changes in the immunological index as well as the clinical symptoms of RA due to the effect of the drug. The collected blood samples will be centrifuged at 3000 rpm at 25 °C for 15 min and then stored at −70 °C in a deep freezer. At the end of the clinical trial, all serum samples will be analyzed simultaneously to confirm any changes in levels of inflammation-related cytokines and chemokines. The items to be analyzed are as follows: inflammatory cytokines (TNF-α, IL-1β, and IL-6), chemokines (IL-8 and MCP-1), and vascular endothelial growth factor.

##### Korean medical diagnostic pattern indicator

We will use the BSQ to classify the Korean medical diagnostic pattern of the patients. Patients with rheumatic diseases generally have a blood stasis pattern. The BSQ consists of 12 items with proven reliability and validity. Each item is rated on a seven-point Likert scale: 1 = disagree very strongly, 2 = disagree strongly, 3 = disagree, 4 = neither agree nor disagree, 5 = agree, 6 = agree strongly, and 7 = agree very strongly. We will convert Likert scores of 1–4 to 0 points and Likert scores of 5–7 to 1 point to calculate the total BSQ scores. The cutoff is 3 points in judging whether a patient has a blood stasis pattern [21].

### Feasibility assessments

To assess the feasibility of a large-scale definitive RCT, in this pilot trial we will assess the process, resources used, and management of the study design as well as the efficacy and safety of the drug [22]. The process assessment looks at whether the eligibility criteria in the pilot study are sufficient or too restrictive; the rates of recruitment, retention, and refusal; and the recruitment time needed for the main study. The resource assessment will consider the length of time to complete the measurement form and whether the researchers and the center have the capacity to conduct the trial. The management assessment will focus on data-optimization issues including data collection, recording, entry, and storage in the center.

### Sample size

No previous clinical trials have evaluated the effect of *geonchildan* for patients with RA. A formal sample size calculation was not performed because this study will evaluate the feasibility of and inform the sample size calculations of subsequent definitive RCTs. A sample size of 12 is the recommended minimum number per group to be considered for pilot studies [23]. Therefore, a sample size of 15 per group and a total number of 30 will be included considering a possible 20% dropout rate.

### Data management

To promote data quality, data collection procedures will be conducted in compliance with the approved protocol and the trial’s standard operating procedures. Participants will be encouraged to complete the study. The participants will be thoroughly briefed on the process of the study and notified of their visit schedules via phone calls or text messages. After the end of the trial, the data will be double entered and matching will be conducted after inconsistent data have been compared to the original case report form. When the data are matched, a data-clarification form will be completed and validated; the resolution will be reflected in the data.

### Statistical analysis

All statistical analyses will be conducted by a statistician blinded to group allocation with the SAS package. The level of significance will be set at 5% in a two-tailed test. Multiple imputation methods will be used to replace missing values.

The analysis set will consist of a full analysis set, a per protocol (PP) set, and a safety set. The full analysis set will include all randomized participants who have received any study treatment and had at least one assessment after treatment. The PP set will include only participants who have completed the treatment according to the protocol. The minimum compliance rate of participants receiving the investigational drugs for the PP set is 80%. The safety set will include any participants who were randomly assigned and have received at least one investigational drug. The main analysis will use the full analysis set. It will be compared to the PP analysis as a sensitivity analysis. An interim analysis will not be performed.

For the descriptive analysis, Student’s *t*-test or a Wilcoxon rank sum test for continuous data and a chi-squared test or Fisher’s exact test for categorical data will be performed.

For a confirmatory analysis, an analysis of covariance will be conducted to compare the mean differences in DAS28 scores between the two groups by substituting each group and the 12-item BSQ score for fixed factors, points acquired at baseline for covariates, and points assessed at week 12 for dependent variables. The secondary outcomes will be analyzed following the same methodology used for the primary outcome. A repeated measures analysis of variance will be used to identify any trend changes.

A safety assessment will be performed for all AEs occurring during the study period. The incidence of AEs, AEs leading to withdrawal, and serious AEs will be summarized by group and analyzed using Fisher’s exact test or a chi-squared test.

### Data monitoring

Data and safety will be monitored regularly by the Korea Institute of Oriental Medicine to control the quality of the data. The clinical research associate will monitor the written informed consent documents, protocol compliance, data quality, and overall trial progress during the study period. The study will proceed until 30 participants are randomly assigned and have completed the study.

### Adverse events

At every visit during the treatment period, the researchers will record AEs based on the Rheumatology Common Toxicity Criteria [24]. A causal relationship between the investigational drugs and AEs will be assessed using a six-grade scale (1 = definitely related, 2 = probably related, 3 = possibly related, 4 = probably not related, 5 = definitely not related, and 6 = unknown), and the severity of the AEs will be scored using a four-point Likert scale (mild, moderate, severe, or life-threatening event). Pruritus, which is a known frequent allergic reaction to *Rhus verniciflua* Stokes, will be carefully monitored [24, 25]. If AEs due to the investigational drugs occur, researchers will alter the dose or frequency according to the standard operating procedures.

## Discussion

This is the study protocol of a randomized, double-blind, placebo-controlled clinical pilot trial assessing the effect of *geonchildan* on patients with active RA. This study wiill evaluate the efficacy and safety of *geonchildan* compared to placebo after 12 weeks of treatment and assess the feasibility of a large-scale RCT. We will administer *geonchildan* to patients with active RA, even if they have used DMARDs for at least 3 months, and evaluate the reduction in the active state by measuring DAS28 and the ACR response and whether an additional prescription of other DMARDs or biological agents was necessary after the 12-week oral administration of *geonchildan*.

RA can be characterized by blood vessel disease, such as angiogenesis or new blood vessel growth, as well as synovial tissue leucocyte ingress [26, 27]. In particular, the formation of new microvessels within the synovium, which provide a nutritional supply for the proliferation of the synovial pannus, are known as major features of blood vessels in RA [27]. These pathological changes in blood vessels in RA are similar to those in blood stasis, which is a traditional pattern-identification diagnosis [28, 29]. Abnormal new blood vessel growth and a layer of fibrovascular tissue, called a pannus, are major characteristics of blood stasis. *Rhus verniciflua* Stokes, a major compound of *geonchildan*, has been used to resolve blood stasis and treat inflammatory arthritis [30]. Thus, we developed *geonchildan*, an extract of *Rhus verniciflua* Stokes, to reduce RA and symptoms associated with blood stasis.

To our knowledge, no trial has so far assessed the efficacy and safety of *Rhus verniciflua* Stokes in patients with active RA. Hence, we specifically designed a small-scale pilot study to assess the feasibility of the process, resources used, and management of the study as well as to estimate the treatment effect and safety of *geonchildan*. If the superiority of *geonchildan* versus the placebo is demonstrated and 12-week of *geonchildan* is tolerable for patients with active RA, this study could form the foundation for a large-scale clinical trial.

### Trial status

The study was launched on 24 March 2016. The most recent version of the protocol (version 2.0) was approved by the institutional review board on 17 March 2017. Recruitment is expected to be complete by July 2018. The results of this study will be reported in December 2018.

## Additional file

Additional file 1: SPIRIT checklist. (DOCX 49 kb)

## Abbreviations

ACR: American College of Rheumatology
AE: Adverse event
BSQ: Blood Stasis Questionnaire
DAS28: Disease Activity Score for 28 joints
DMARD: Disease-modifying antirheumatic drug
ECG: Electrocardiography
ESR: Erythrocyte sedimentation rate
EULAR: European League Against Rheumatism
KHAQ: Korean Health Assessment Questionnaire
MFDS: Ministry of Food and Drug Safety
NSAID: Nonsteroidal anti-inflammatory drug
PP: Per protocol
RA: Rheumatoid arthritis
RCT: Randomized controlled trial
VAS: Visual analogue scale
